# The Role of the Stimulus in Olfactory Plasticity

**DOI:** 10.3390/brainsci13111553

**Published:** 2023-11-06

**Authors:** David M. Coppola, Johannes Reisert

**Affiliations:** 1 Biology Department, Randolph-Macon College, Ashland, VA 23005, USA; 2 Monell Chemical Senses Center, Philadelphia, PA 19104, USA; jreisert@monell.org

**Keywords:** odor, enrichment, deprivation, activity dependence

## Abstract

Plasticity, the term we use to describe the ability of a nervous system to change with experience, is the evolutionary adaptation that freed animal behavior from the confines of genetic determinism. This capacity, which increases with brain complexity, is nowhere more evident than in vertebrates, especially mammals. Though the scientific study of brain plasticity dates back at least to the mid-19th century, the last several decades have seen unprecedented advances in the field afforded by new technologies. Olfaction is one system that has garnered particular attention in this realm because it is the only sensory modality with a lifelong supply of new neurons, from two niches no less! Here, we review some of the classical and contemporary literature dealing with the role of the stimulus or lack thereof in olfactory plasticity. We have restricted our comments to studies in mammals that have used dual tools of the field: stimulus deprivation and stimulus enrichment. The former manipulation has been implemented most frequently by unilateral naris occlusion and, thus, we have limited our comments to research using this technique. The work reviewed on deprivation provides substantial evidence of activity-dependent processes in both developing and adult mammals at multiple levels of the system from olfactory sensory neurons through to olfactory cortical areas. However, more recent evidence on the effects of deprivation also establishes several compensatory processes with mechanisms at every level of the system, whose function seems to be the restoration of information flow in the face of an impoverished signal. The results of sensory enrichment are more tentative, not least because of the actual manipulation: What odor or odors? At what concentrations? On what schedule? All of these have frequently not been sufficiently rationalized or characterized. Perhaps it is not surprising, then, that discrepant results are common in sensory enrichment studies. Despite this problem, evidence has accumulated that even passively encountered odors can “teach” olfactory cortical areas to better detect, discriminate, and more efficiently encode them for future encounters. We discuss these and other less-established roles for the stimulus in olfactory plasticity, culminating in our recommended “aspirations” for the field going forward.

## 1. Introduction

Plasticity, as applied to a nervous system, refers to the system’s ability to be altered by experience and is a concept intertwined with classical nature–nurture debates. William James (1842–1910) is usually credited with being the first to use the term plasticity with respect to behavior while Jean Demoor (1867–1942), a Belgian anatomist, was apparently the first to use the term with respect to neurons [1,2]. Victor Malacarne (1744–1816), the Italian pioneer of cerebellar anatomy, is said to have planned the first nature–nurture experiment proposing to compare the cerebellums of trained and untrained dogs [3]. Alas, it is unclear if he ever followed through on his plans. The German Bernhard von Gudden (1824–1886), studying the plasticity of the olfactory system—the topic of this review—carried out among the first sensory deprivation experiments, heralding 20th-century neuroscience’s preoccupation (now considered passé) with differentiating the effects of nature and nurture [4]. But, the search for and explication of plasticity mechanisms still dominates the field of neuroscience.

Here, we review the classical and contemporary literature dealing with the role of the stimulus in olfactory plasticity. The work focuses on plasticity engendered by manipulating the quantity and quality (type and complexity) of the stimulus. The voluminous literature renders it impractical to be exhaustive, though we believe our review to be the most inclusive to date. Two necessary but regrettable exclusions are papers dealing with non-mammals and the burgeoning literature on component interactions in odor mixtures.

## 2. The Olfactory System

**Figure 1:**
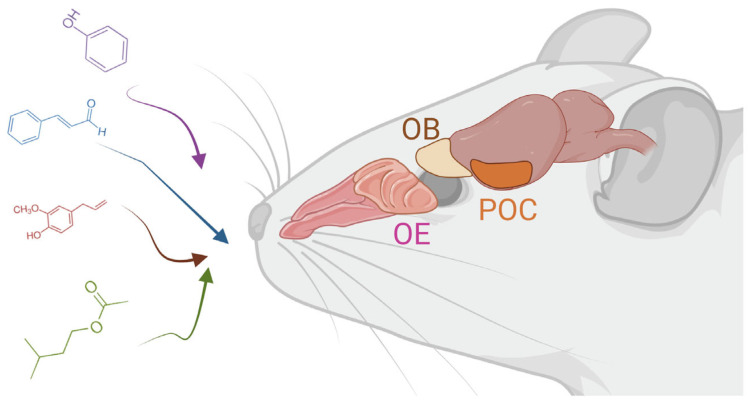
Overview of the olfactory system showing three anatomical structures that are commonly studied in deprivation and enrichment experiments. OE: olfactory epithelium; OB: olfactory bulb; POC: primary olfactory cortex. Created with BioRender.com.

The mammalian olfactory system consists of a sensory epithelium, positioned in the dorso-caudal region of the nasal cavity. Olfactory sensory neurons (OSNs) from this epithelium send coded information to central structures that give rise to odor perception (Figure 1). Unique among sensory neurons, new OSNs are produced throughout life as mature OSNs die. As OSNs mature, they send axons to telencephalic structures, the olfactory bulbs [5]. In the bulbs, OSNs form synapses with the mitral and tufted cells, in neuropil structures called glomeruli. A large mammalian gene family codes for olfactory receptors (ORs), such that each OSN expresses one of ~1000 genes in the mouse [6]. OSNs expressing a particular OR typically converge onto only one medial and one lateral glomerulus in each bulb [7]. Once it reaches the bulbs, odor information is processed by this laminar structure through its elaborate direct and indirect pathways [6]. Periglomerular and granule cells are the key inhibitory interneurons of the bulb. These cells are replaced by progenitor cells arriving in the bulb from a rostral migratory stream.

Once olfactory information reaches the mitral and tufted (M/T) cells of the bulb, it is transmitted to a set of central targets, the primary olfactory cortex, which includes the piriform cortex, the entorhinal cortex, the amygdala, and the accessory olfactory nucleus. In these central areas, odor is presumed to gain its perceptual and emotional qualia [6].

## 3. The Effects of Stimulus Deprivation

**Figure 2:**
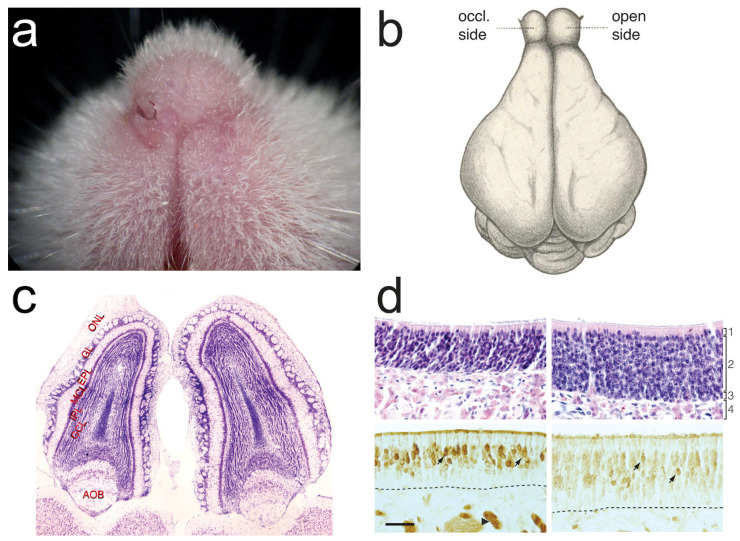
UNO’s structural effects. (**a**) Adult mouse with left neonatal UNO. (**b**) Brain illustration of an adult rabbit that had neonatal UNO [4]. Dorsal view is reflected right/left; occl = occluded). (**c**) Olfactory bulbs in the horizontal section from an adult mouse that had neonatal UNO. All layers of the bulb on the occluded side (**left**) are thinner than those of the open-side bulb (**right**). ONL = olfactory nerve layer; GL = glomerular layer; EPL = external plexiform layer; MCL = mitral cell layer; IPL = internal plexiform layer; GCL = granule cell layer; AOB = accessory olfactory bulb. (**d**) Sections of olfactory mucosa from an adult mouse that had neonatal UNO. Open side (**right**); occluded side (**left**); top row is H&E stain; bottom row is OMP immunolabeling (arrows are mature OSN cell bodies); epithelial layers: 1 = sustentacular layer; 2 = olfactory receptor cells; 3 = basal cell layer; 4 = lamina propria. Modified from [10].

Unilateral naris occlusion (UNO) has been the dominant method for creating an odorant impoverished state in animal studies of olfactory plasticity. Deodorizing environmental air has rarely been attempted, likely because contamination by odors emanating from the subject (e.g., urine, feces, skin secretions) is unavoidable. Other manipulations, like selective chemical poisoning of OSNs (e.g., zinc sulfate lavage) and genetic knock-out of components of the transductory cascade have failed to supplant UNO in most deprivation studies because of their side-effects, non-specificity, and/or failure to reliably block most sensory inputs [8,9]. Consequently, our review of odor deprivation effects will be limited, largely, to those caused by UNO (Figure 2).

A large body of work documents the many consequences of UNO on the ipsilateral olfactory pathway and, to a lesser extent, on the contralateral pathway. Early experiments usually focused on UNO’s detrimental and age-dependent effects. The former refers to the loss of capabilities or components and is consistent with a “use-it-or-lose-it” perspective—for example, a loss of OSNs. The latter refers to results that occur only or most prominently during a sensitive developmental window and support the existence of an olfactory critical period, like that observed in the developing visual system [11]. Unfortunately, an olfactory critical period has been assumed in many studies, leaving the question of a sensitive developmental window untested for some reported effects of deprivation and enrichment.

More recent research has focused on life-long vulnerability to deprivation, particularly on neurogenesis, and compensatory responses. By compensation, we mean responses to deprivation, which appear to counteract the loss of input signal—for example, the up-regulation of the OSN transductory pathway that could reasonably be expected to increase sensitivity (see below). Both the deleterious and compensatory effects of deprivation will be reviewed briefly, starting with findings from the periphery and working centrally. However, as will be revealed in what follows, the deleterious/compensatory dichotomy is an oversimplification, which, while aiding our discourse, (perhaps) fails to capture the complex network of responses to manipulations of the stimulus in olfaction.

### 3.1. Deleterious Effects of UNO

#### 3.1.1. Mucosa and OSNs

In newborn rats, ipsilateral olfactory mucosal metabolism, measured by succinate dehydrogenase histochemistry, is markedly reduced within two days UNO [12]. In rabbits, mice, and rats, UNO causes a decrease in the thickness of both the ipsilaterally respiratory and olfactory mucosa which is more pronounced if occlusion occurs soon after birth [13,14,15]. In rats, neonatal UNO causes a decline in mitotic rate in the olfactory and respiratory epithelium [14,16,17]. This effect on the mitotic rate can be reversed by reopening the occluded naris [18]. In the rabbit, a decrease in OSNs on the occluded side has been reported [15]. Despite the difference in mucosal depth, in the patent and occluded nasal passages, the abundance of mature OSNs has been shown in some rodent studies to be unaffected by nostril occlusion [13,14]. In other rodent studies, a decrease in OSNs has been found on the occluded side compared to the open side [19,20]. In yet another study, UNO caused both a decrease in OSN proliferation and an increase in apoptosis in the occluded fossa [17]. In a related finding, an activity-dependent histone variant was discovered that appears to decrease OSN longevity [21]. Ten days of UNO in adult wild-type mice increased the abundance of this histone variant, portending shorter longevity for OSNs on the occluded side of the nasal cavity. This finding provides a mechanism for odorant deprivation-related reductions in mature OSNs, but given the contradictory results, the issue of deprivation’s effect on the abundance of mature OSNs is unresolved.

Rats that underwent UNO as neonates showed an expansion of olfactory mucosa on the occluded side at 60 days of age [22]. In a related study, turbinates appear less robust compared to their open-side counterparts in three-to-four-week-old mice that had undergone UNO on the day after birth [23].

A mature body of research has established OSN activity as a key player in the expression of axon guidance molecules. These studies have consistently shown a dearth of guidance molecules in the occluded-side mucosa of developing rodents that had undergone UNO soon after birth [24,25,26].

Finally, a growing list of studies has examined the effects of deprivation on gene expression in OSNs either by bulk analysis using microarrays or RNA seq [27,28] or using single-cell methods [29,30]. Since none of these results fit unambiguously under the “deleterious” umbrella, we will discuss them below under the heading “Compensatory Effects of Deprivation”.

#### 3.1.2. Olfactory Bulb

It has been known since the 19th century that UNO performed on the neonatal rabbit prevents the ipsilateral olfactory bulb from reaching its normal adult size, a finding that has been replicated in a few different species [13,31,32]. The failure of the developing ipsilateral olfactory bulb to reach its normal size after UNO is due, partly, to reductions in the thickness of the glomerular and external plexiform layers [33]. More recently, the effect of UNO on ipsilateral glomerular size has been demonstrated repeatedly in P2-odorant receptor reporter mice [19,34,35,36]. In a recent study of the olfactory “critical period”, mice underwent UNO on the day of birth, after which the occluded naris was reopened in different groups at various time points thereafter. The presynaptic marker vGlut2 and the postsynaptic marker vGlut1 where then measured in all subjects at postnatal day 21 (PND21) by immunochemistry. Consistent with the presence of a critical period, mice that had their naris reopened at PND6 had normal synaptic marker levels in the glomeruli on the formerly occluded side, while mice with their naris reopened at later time points had reduced levels [36].

The granule cell layer in the ipsilateral bulb displays the most dramatic decline after the UNO of any lamina [37]. Classic tritiated thymidine and bromodeoxyuridine techniques have shown that the loss of granule cells from the bulb on the occluded side is predominantly due to a decrease in cell survival, not a decrease in progenitor cell supply from the rostral migratory stream [38,39].

While these anatomical effects of UNO can take weeks to detect, metabolic changes can appear rapidly in many cases: For example, in the olfactory bulb on the occluded side, 2-deoxyglucose uptake and Krebs-cycle enzymes decline in a matter of days following UNO [40,41]. Following UNO, rapid declines in protein synthesis (measured by radiolabeled amino acid uptake), and in gene expression (measured by in situ hybridization) on the occluded side have long been known [42].

Neurochemically, the rate-limiting enzyme in dopamine synthesis, tyrosine hydroxylase, declines in the ipsilateral bulb within days of UNO, a deficit that can be reversed by reopening the occluded naris [43,44,45]. Consistent with this finding, the tyrosine hydroxylase and dopamine content of juxtaglomerular cells, the predominant dopaminergic neurons of the bulb, decline after UNO, olfactory nerve axotomy, or chemical lesion of the olfactory mucosa [44,46,47]. UNO leads to a down-regulation of β_1_ and β_2_-adrenergic receptors [48] but apparently does not affect norepinephrine receptors [49]. Glutamate receptors, as a group, are apparently not markedly affected by UNO. However, in the bulb’s external plexiform layer, GluR1-expressing short-axon neurons are reduced in number on the occluded side compared to age-matched controls [50].

UNO affects bulbar neurotrophic factors and neuromodulators. In the rat, nerve growth factor receptors are more numerous on the occluded compared to the open side 19 and 60 days post neonatal naris occlusion [51]. Brain-derived neurotrophic factor (BDNF) increases initially and later decreases on the side of occlusion [52]. Insulin receptor kinase decreases on the occluded side. This is interesting given that this receptor and its ligand, along with BDNF, modulate ion channels in certain bulbar neurons [53,54,55]. Finally, the mitogen-activated protein kinase/extracellular signal-regulated kinase (MAPK/ERK) pathway, a fundamental cellular-signaling cascade, is also diminished on the occluded side after UNO [56].

The deleterious effects of deprivation on the bulb have been shown at the level of circuits: in rats, 21 days of UNO, beginning the day after birth, induce the enhanced inhibition of M/T cells in response to the paired-pulse stimulation of the lateral olfactory tract [57,58], an effect which is NMDA receptor-mediated [59]. In adult rats, 1–2 months or 12 months of UNO increased the fraction of M/T cells from the occluded-side bulb that respond to multiple odorants, consistent with decreased discrimination [59]. And, in immature rats, as little as 15 min of naris occlusion induced a decoupling of M/T responses from the respiratory cycle [60]. In another study, early naris occlusion delayed the development of ipsilateral mitral cells and abrogated the normal switch in membrane conductance and coupling coefficients that constitute the normal maturational change from electrical to chemical bulbar synapses [61]. Nevertheless, electrophysiology has, mostly, failed to show differences in the circuit properties of the occluded-side bulb after UNO, which might be expected from the major structural and biochemical effects reviewed thus far [62].

Olfactory bulb size in humans, measured by MRI, is positively correlated with psychophysical smell test scores both in head-trauma patients as well as in typical adults and young people [63,64,65]. Patients with the most severe chronic rhinosinusitis (analogous to deprivation) tended to have the smallest olfactory bulb volumes and poorest olfactory performance, suggesting this relationship is causally linked and plastic over one’s lifetime [66,67]. A longitudinal study of patients with chronic rhinosinusitis prescribed the standard treatment regimen reported an increase in bulb size with a decrease in odor thresholds [68]. This body of clinical results suggests that the magnitude of peripheral input may affect cell survival in the olfactory bulb, consistent with some rodent studies [69].

#### 3.1.3. Cortex

At the cortical level, piriform layer 1b’s thickness and the size of its semilunar cell dendrites were reduced on the ipsilateral side of rats with UNO from PND1 through PND 30 [70]. NMDA receptor NR2B expression and phosphorylated CREB declined in the piriform after five days of UNO, an effect that was reversible ten days after the occluded naris was reopened [71]. Additionally, in a rat piriform cortical slice preparation, neonatal UNO delays the normal increase in the ratio of AMPA to NMDA receptors at primary sensory synapses [72]. Notably, this ratio is not affected in associational synapses on pyramidal neurons by UNO [72]. In related research, field potentials from anterior piriform show occluded-side response depression evoked by the stimulation of cortical afferents at PND1 but not at PND30 [73]. However, evoked potentials in the piriform association fiber of UNO rats were enhanced ipsilaterally in both PND1 and PND30. Consistent with these findings, an fMRI study in humans showed that seven days of bilateral olfactory deprivation by naris obstruction caused a reversible decrease in odor response signal from the anterior piriform cortex along with a surprising increase in signal from the orbitofrontal cortex (see below) [74].

In other central structures, UNO from PND1-20 caused a decline in 2-deoxyglucose uptake in the rostral anterior olfactory nucleus [75]. To the best of our knowledge, the effect of UNO on other central olfactory structures (e.g., amygdala and entorhinal cortex) is an open question.

#### 3.1.4. Neurogenesis

Olfaction, arguably, has the greatest plasticity of any sensory system. As alluded to previously, the olfactory epithelium produces a constant supply of new OSNs. New interneurons, originating from a niche in the sub-ventricular zone (SVG), make their way to the bulbs along the rostral migratory stream, also in a continuous process. The bulb’s supply of newborn neurons differentiates mostly into juxtaglomerular and granule cells, both inhibitory interneurons. In the epithelium, precursor cells, near the basal lamina, differentiate into new OSNs that send dendrites to the epithelial surface. Meanwhile, these inchoate OSNs grow axons that migrate to the olfactory bulb glomeruli where they form functional connections with 2nd-order neurons [76]. This lifelong process underlies the replacement of mature neurons with newborn neurons in cycles with periods of perhaps two months [5,77].

As noted above, UNO decreases the birth rate of OSNs [14,16,17]. Thus, OSN proliferation appears to be stimulus-dependent. In contrast, UNO does not appear to influence neurogenesis in the SVG [78]. However, the survival and functional integration of adult-born neurons reaching the bulb are reduced by UNO [79]. In the enrichment section of this review, we will come back to this topic. However, a full review of the expansive literature on the role of adult-born neurons in olfaction is beyond our intended scope.

#### 3.1.5. Behavior

Despite the many deleterious effects documented above, we know of only two studies, at the behavioral level, showing an olfactory deficit following deprivation [36,80]. In one study, human subjects were randomly assigned to one of two modes of deprivation: Wearing a “filtering mask”, or a nasal appliance designed to shunt inhaled air directly toward the throat and away from the olfactory mucosa. There was also a group of un-instrumented controls. Experimental subjects were asked to wear their assigned device for six to eight hours per day for 14 days. Each subject’s olfactory acuity was tested both before and after the deprivation period. Subjects in the nasal appliance group but not the masked group displayed a modest increase in threshold (decrease in acuity) after deprivation but no change in odor identification. Interestingly, the mask-wearing group showed a small but significant increase in odor identification, perhaps suggesting a compensatory process.

In a recent mouse study investigating the olfactory critical period (see above), subjects underwent UNO on the day of birth followed by naris reopening at PND 6 or PND 10 [36]. Subsequently, these groups were tested at 6 weeks of age with their normal naris plugged, forcing them to use their formerly occluded naris in tests of olfactory acuity. The investigators used a standard habituation/dishabituation paradigm for the behavioral tests. Consistent with the critical period concept, only the PND10 reopened group showed deficits in olfactory acuity, including tests of the discrimination of the enantiomers of carvone.

### 3.2. Compensatory Effects

#### 3.2.1. Mucosa and OSNs

Adenylate cyclase type III (AC_III_), a key component of the olfactory transductory pathway, and PDE4A, a non-ciliary phosphodiesterase that modulates transduction, increase in concentration ipsilaterally in response to UNO [81]. Subsequently, microarray analysis with PCR validation was used to confirm and extend these immunolabeling results [27]. The adult olfactory epithelium transcriptomes of untreated mice were compared to epithelia from the ipsilateral and contralateral nasal cavities of mice that had neonatal UNO. Genes implicated in olfactory reception, transduction, and transmission, including numerous ORs, were up-regulated in the deprived-side olfactory mucosa. Opposite effects accrued on the non-deprived-side mucosa, compared to controls. These protein-level and bulk transcriptomic-level results were among the first to suggest that the odorant environment can trigger a compensatory response in OSNs, a conclusion that has been confirmed and extended by recent studies at the single-cell level [29,30]. These single-cell RNA sequencing (scRNAseq) studies have uncovered an unexpected diversity of transcriptomes that are characteristic of the OR carried by an individual OSN. Importantly, UNO caused functional gene expression to change in a direction that appears to compensate for the low level of OSN activity created by UNO [29].

**Figure 3:**
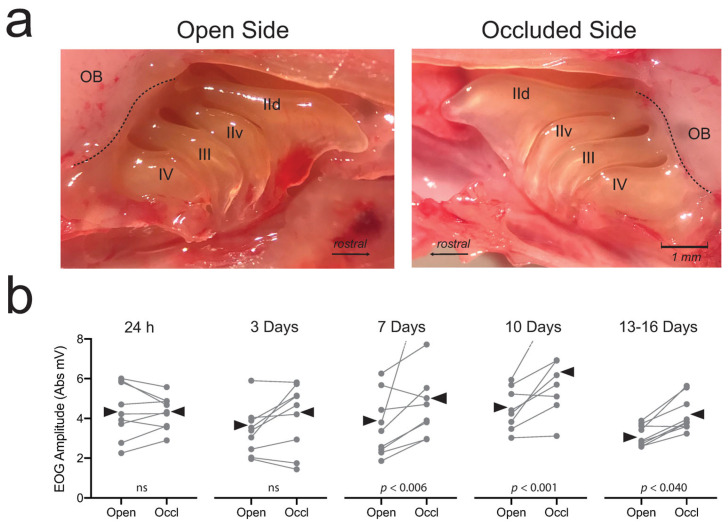
(**a**) Micrographs of unilaterally naris-occluded mouse nasal cavity. Midsagittal views of turbinates from the open side and occluded sides of a mouse that underwent UNO more than one year prior. Roman numerals denote turbinates. Observe the similar gross anatomical appearance of the open and occluded sides. (**b**) Absolute values of EOG amplitudes (filled circles) evoked by stimulation with a 0.1% concentration of isoamyl acetate are plotted on the ordinant. Subjects wore a nasal plug for the designated durations. Open and occluded (Occl) side responses from a given mouse are connected by lines. Arrow heads = means. Sidlak’s multiple comparison test probabilities for differences between open and occluded side means or shown. Modified from [86].

At the physiological level, electroolfactogram (EOG) recordings have been used to investigate compensatory responses to olfactory deprivation. The EOG is an ensemble recording of generator potentials from OSNs measured at the surface of the olfactory epithelium [82,83]. In one study, EOGs were recorded from identical locations on the ipsilateral and contralateral mucosae of UNO mice [84]. Consistent with the transcriptomic and protein findings already mentioned, EOG amplitudes from the occluded side of UNO mice were greater for a given odorant and concentration than those from the open side. These results imply that odor-deprived OSNs develop larger generator potentials or that more OSNs are responsive to a particular odorant, or both. Subsequently, these results were replicated in adult mice (Figure 3) [85,86].

#### 3.2.2. Olfactory Bulb

One of the first studies of the effects of UNO on primary and secondary synapses in the olfactory bulbs by Tyler and colleagues used the whole-cell voltage-clamp in a rat slice preparation [87]. Two weeks of UNO, starting on PND2, increased the quantal content and the probability of transmitter release at first-order olfactory synapses in the occluded-side bulb. Moreover, the effects of naris occlusion were manifest within three days of naris occlusion. By contrast, a study in adult mice transgenic for a calcium fluorophore, allowing imaging of the activity in the first order, found a different result [88]. Four weeks of naris occlusion caused a decrease in the odorant-evoked synaptic release on both the open side and occluded side of the UNO group. However, the voltage-clamp result—compensation—was further supported by the immunolabelling of the vesicular glutamate transporter and two glutamate receptor subunits, which demonstrated that naris occlusion caused an increase in these synaptic components at ipsilateral olfactory synapses. In addition, spontaneous and evoked voltage-clamp activity in M/T neurons showed that UNO also strengthens 2nd-order synapses of the bulbar circuit. This could explain previous observations in UNO rats that the size and intensity of 2-deoxyglucose foci are increased in the occluded-side glomeruli following the opening of the occluded naris [62]. In a follow-on study, M/T cells were more excitable after 1–2 months and 12 months of UNO, suggesting that stimulus deprivation may increase odor detectability while compromising odor discrimination [89]. Recently, George and colleagues added to these findings by showing in adult mice that 30 days of UNO leads to mitral cells from the occluded bulb with broadened action potentials [90]. This change could increase the synaptic release from these cells, consistent with Tyler and colleagues, thus representing a clearly compensatory response to deprivation [87]. George and colleagues also found changes in mitral cell myelination on both the occluded and non-occluded bulbs compared to controls, which cannot neatly fit into the compensatory or deleterious dichotomy.

Olfactory bulb neurotransmitter systems also show compensation: for example, following UNO, there is a decrease in occluded-side bulb dopamine that triggers a >30% increase in dopamine D_2_ receptors even after correcting for laminal shrinkage [91]. Analogously, an increase in ipsilateral bulb norepinephrine triggers a decrease in norepinephrine receptors [92]. In another example of compensation, the UNO-induced ipsilateral decrease in glomerular neuropil and mitral cell dendritic arbors leads to a more uniform distribution of synaptophysin [92]. Finally, the UNO-induced loss of occluded-side granule cells is partially compensated for by the increased excitability of surviving granule cells [39]. These findings could explain how the occluded-side olfactory bulbs of animals subjected to UNO appear to maintain normal function.

#### 3.2.3. Olfactory Cortex

Wu and colleagues have extended the search for the compensatory effects of stimulus deprivation to the human olfactory cortex [74]. Subjects were asked to wear nose plugs bilaterally for 7 days and were followed through a subsequent 7-day recovery period. Comparisons between fMRI scans before and after deprivation showed reduced odor responses in the posterior pyriform cortex—the largest area of the olfactory cortex—but enhanced responses in the orbitofrontal cortex—a higher-order olfactory center. Examinations of recovery scans showed that these effects were reversible a week after the nasal plugs were removed. Given the absence of changes in behavioral acuity, the authors interpreted the orbitofrontal cortex-enhanced signal as a compensatory mechanism.

#### 3.2.4. Behavior

As described in the previous section, the effects of a week of odorant deprivation had no consistent effect on olfactory psychophysical tests in human subjects despite the fMRI signal modifications in the piriform and orbitofrontal cortex [74]. Consistent with this finding, rats that had undergone either acute or chronic UNO and contralateral bulbectomy were able to detect odors presented at low concentrations [93,94]. Moreover, like-treated newborn mice used odor cues to find their mother’s nipples and quickly navigate back once displaced from the nest [95]. In a similar study, adult mice underwent UNO as neonates and contralateral bulbectomy as adults. Once the occluded naris was reopened, these subjects outperformed unilateral bulbectomized mice *without* contralateral UNO on two different tests of olfaction [96]. One explanation for these observations is that the nasopharyngeal canal, present in many mammals, including mice, provided a pathway for odorants to reach the occluded-side mucosa from the open-side naris [93]. Of course, it is also likely that odorants gain access to the occluded mucosa by a retronasal route. Whatever the route of odorants to reach OSNs in these UNO/contralateral bulbectomy mice, their superior olfactory acuity provides compelling evidence for compensatory responses to deprivation.

### 3.3. Theory

Meisami can be credited for the rediscovery, in the 1970s, of the UNO technique [31]. Consistent with the contemporary zeitgeist, he interpreted his observations of atrophic responses in the occluded-side olfactory system as examples of the activity dependence of normal neural development [97]. In this paradigm, suggested by Hebb, neural activity strengthens useful synaptic connections and a lack of activity eliminates useless synaptic connections [33]. However, Hebb’s postulate is inadequate, at least by itself, to account for the phenomenology of UNO. More specifically, the idea that experience serves an “instructive” role in the construction of the bulbar odor map has been undermined by contrary evidence [98]. This evidence includes the use of mouse strains lacking transductory cascade components, which have been used to show that the target guidance of OSN axons does not depend on odorant-driven activity, though spontaneous activity is required [6,99]. From another perspective, parallels between activity-dependent phenomena in other sensory systems and those that accrue to UNO seem inapt. Consider that the marked reduction in bulb size that is caused by UNO has no parallel in other sensory systems deprived of their target stimulus. In the visual system, for example, dark-rearing does not cause the atrophy or cell loss observed in the olfactory bulb upon UNO either in the neonate or adult [100]. On the contrary, dark-rearing has modest effects on the appearance and circuit properties of visual pathways [63]. Stimulus deprivation in the somatosensory system has, for the most part, similarly subtle effects at the gross anatomical level [101].

By contrast, the transcriptomic, proteomic, and electrophysiological results are consistent in support of the fact that the conclusion that the olfactory periphery responds to deprivation in a compensatory manner. For example, the implication of the increase in abundance of the transductory enzyme AC_III_ in response to deprivation seems obvious: deprivation leads to an increase in cAMP, which triggers OSNs to initiate action potentials [102,103]. Thus, stimulus deprivation begins a biochemical cascade that leads to an increase in OSN “gain”, which preserves function under conditions of stimulus scarcity [90]. The reported gain in primary and secondary synapses in response to deprivation is inconsistent with Hebb, being more in line with the theory of homeostatic plasticity [87,90,104]. Finally, the clearest evidence of compensation is offered by the observation that mice relying exclusively on their occluded olfactory system show superior performance to controls on behavioral tests of olfaction [94].

## 4. Stimulus Enrichment

### 4.1. Introduction

Herein we use the term enrichment promiscuously to include any provision of extraneous odorant by an experimenter for the purpose of studying activity-dependent plasticity. Unlike stimulus deprivation, which can be described in absolute terms—if not absolutely achievable experimentally—enrichment can only be operationally defined. Moreover, there is no consensus as to what qualifies as olfactory enrichment. One impediment to standardization is that we have very little knowledge of the statistics of odor environments, even those inhabited by domesticated animals, much less the obviously more complex odorant milieus of their wild counterparts. In most cases, we simply do not know what odorants are present in the environment, at what concentrations, and how odor environments change across time and space. One, then, might rightly ask: if we do not know what a typical odor environment consists of, how can we create an enriched one? Adding to the problem, animal olfactory systems are exquisitely sensitive, making the task of constructing a complete odor inventory for any environment extremely challenging. In most cases, odor “enrichment” has taken the form of exposure to a single odorant, typically applied at an exceedingly high concentration that is delivered for long durations, either intermittently or continuously. Also, administration protocols are not standardized, giving rise to a myriad of enrichment schedules. Nevertheless, investigators have been little troubled by these considerations, as you will see in the work to be reviewed.

As was the case for the odorant deprivation section above, we will start with the periphery and advance centrally.

### 4.2. Mucosa and OSNs

Perhaps the most surprising account of stimulus-dependent olfactory plasticity is “induction”. This phenomenon was first demonstrated behaviorally (see behavior section below). Wysocki and colleagues showed that human subjects who were initially unable to smell androstenone, a steroid odorant, developed the ability after brief daily exposures that took place over several weeks [105]. By this time, animal anosmia had already been discovered in a series of behavioral genetic studies showing that the NZB/B1NJ and C57BL/6 mouse strains were models of androstenone and isovaleric acid anosmia, respectively [106]. And, like humans, these mouse strains could be induced [107]. The locus (or loci) of induction—olfactory epithelium, olfactory bulb, or cortex—still has not been fully explicated, but electrophysiological and lesion studies, in mice, suggest that the phenomenon is at least partly peripheral [107,108,109]. Mice from NZB/B1NJ and C57BL/6 strains show an increase in EOG responses to androstenone or isovaleric acid, respectfully, upon completion of two weeks of 16 h-per-day exposure [86,107]. In a related phenomenon, feeding juniper berries to rabbit dams during gestation caused their offspring to have larger EOG responses to juniper odorants when tested postnatally compared to controls whose dams were not fed juniper [110,111]. Enhanced EOG responses to an arbitrary odorant have also been reported in rabbit pups immediately after a single brief (5 min) pairing of the odorant with the nipple pheromone 2-methylbut2-enal [112].

A potential mechanism for induction, at least in principle, is suggested by the finding that the stimulus also promotes OSN survival: odorant exposure protects OSNs, in a cAMP-dependent process, from bulbectomy-induced apoptosis [113]. This process should, over time, result in more OSNs with receptors for the enrichment odor. OSN rescue may be afforded by an olfactory-specific histone variant, H2BE, whose expression is activity-dependent and has a direct influence on OSN lifespan [21].

But, not all investigators have found odorant enrichment to have uniformly sensitizing effects on the olfactory periphery. A study that started enrichment in the prenatal period by feeding pregnant mouse dams with heptaldehyde-laced food, continuing this mode of food-based enrichment postnatally until young adulthood, observed a decline in the EOG response by 3 weeks postnatally that was specific to heptaldehyde [114]. These authors also reported a decline in mI7 receptor transcript, the odorant receptor for heptaldehyde, and down-regulation in two members of the olfactory transduction cascade, CNGA2 and ACIII, in the enrichment group [114].

In another study, mice exposed from birth to PND33 to octanal, either on a pulsed or continuous schedule, showed a decline, compared to controls, in their EOG response to this odorant [115]. In the same study, proteomic analysis of enriched and control mucosae revealed a down-regulation of odorant-binding proteins from the enriched group.

A study of adult transgenic reporter mice for the OR M72 showed that intermittent exposure to this receptor’s cognate odorant, acetophenone, for 30 days caused a decline in M72-expressing OSNs [20]. A related study that exposed two different OR reporter strains, MOR23 and M71, to their cognate odorants, lyral and acetophenone, respectively, during the first three weeks of life also found a decline in the abundance of the former receptor but no effect on the latter [116]. However, these authors did find, using patch clamping, a slight increase in sensitivity in MOR23 neurons after enrichment but no change in M71 sensitivity after enrichment. Complicating this picture, quantitative PCR revealed that enrichment with lyral triggered an increase in the mRNA expression of MOR23 and an increase in some but not all olfactory transduction components. Enrichment in the M71 strain did not have a similar effect. Lastly, there was no change in the EOG magnitude for either strain after enrichment. Yet another OR reporter mouse study, focusing on OR m17, found no change in the number of this OSN subtype after constant exposure to its ligand, heptaldehyde, from birth through postnatal day 5 [117]. In a study of MOR29A reporter mice with the cognate odorant vanillin, three ten-minute enrichment sessions per day for 3 days, from PND 2–4, 7–9, or 9–11 did not, for any epoch, lead to a change in the abundance of this type of OSN, consistent with some other studies reviewed [36]. Finally, a study using aversive and appetitive conditioning to the cognate odorant in M71 adult reporter mice showed an increase in M71 OSNs after two training sessions per week for three weeks [118]. Subsequent studies from the same laboratory showed that an extinction paradigm could reverse the increase in M71 OSN number and that odorant fear-conditioning in the parent can affect the OSN number in a subsequent enrichment-naïve generation [119,120].

A growing number of enrichment studies have focused on plasticity in the mucosal transcriptome. Considering near-immediate effects, 30 min of exposure is sufficient to decrease the transcript composition of OSNs cognate for the enriching odorant, an effect which declines within 12 h and disappears after ~24 h [121]. At the other timing extreme, odor enrichment lasting up to 6 months resulted in the modulation of several mouse OR transcripts measured by RNA sequencing [28]. Importantly, intermittent (adulteration of drinking water) but not chronic (pure odorant source in the home cage) enrichment affected mRNA expression with both up- and down-regulation being observed in near equal measures compared to unenriched controls. Two recent studies set a new standard for investigations into OSN transcriptome plasticity with single-cell RNA sequencing (scRNA-seq) to obtain the transcriptomes of hundreds of OSN subtypes [29,30]. While some of the goals and methods of the two studies differ, they are consistent in finding that the transcriptomes of OSN subtypes are remarkably variable and that this variability is partly a function of recent odor-driven activity. Indeed, odor enrichment causes the modulation of several genes that decrease or increase the responses of OSNs, presumably for the purpose of adapting them to a changing odor milieu.

### 4.3. Olfactory Bulb

Studies dating back half a century posed the question: does single odorant enrichment affect the structure of olfactory bulbs? Indeed, continuously exposing rats to single odorants at high concentrations from PND14 for up to 2 months led to changes in the shape and shrinkage in the size of select mitral cells compared to unenriched controls [122]. This study led to a spate of additional studies that form part of the lore of olfactory plasticity research but left the central issue unsettled with some investigators reporting increases and others reporting decreases in mitral cell number and size [123,124,125,126].

Moving ahead to the contemporary era of transgenic reporter mice and elaborate enrichment paradigms, there is still no consensus as to the effects of enrichment on the bulb. In one study, mice carrying a rI7 → 71 reporter transgene that were “conditioned” to the cognate odorant octanal by associating it with their dam’s nipple showed the accelerated refinement of rI7 → M71 glomeruli but no change in glomerular size [127], while mice that were passively exposed to octanal did not differ from controls in the timing of glomerular refinement. In a related study of adult M71 transgenic reporter mice, fear conditioning and conditioned place preference caused cognate odor-trained subjects to form larger M71 glomeruli (see Mucosa section) [118]. This laboratory has subsequently shown that extinction training reverses the olfactory fear-conditioning-based increase in glomerular size [119] and, more amazingly, that parental olfactory experience increases glomerular morphology in subsequent generations, presumably through epigenetic processes [120].

An increase in glomerular size was also reported in a study that fed cognate odorants for ORs M71 and M72 transgenic reporter mice to dams during gestation and lactation and then studied the offspring [114,128]. The results were that unenriched mice and those enriched with noncognate odorants (for their transgenic OR) had normal-sized glomeruli. Yet another study used double knock-in reporter mice for the I7 and M72 ORs and continuously enriched mice with one of the cognate odorants either from PND0 to PND20 or PND20 to PND40. Only the younger cohort developed smaller supernumerary glomeruli and only in those glomeruli corresponding to the cognate enrichment odorant [129]. Thus, the effects of enrichment on glomerular morphology were both odorant and age-specific. A related study in mI7 and M72 reporter mice examined constant enrichment with cognate odorants between 5 and 10 days postnatally [117]. Ten days after enrichment was ended, supernumerary glomeruli were observed for mI7 but not M72 mice. Finally, in a recent study of the olfactory critical period (see above) using MOR29A reporter mice, three ten-minute vanillin enrichment sessions per day for three days were enough to cause an increase in cognate glomerulus size but only if performed before PND7 [36]. Taken together, this array of disparate findings befuddles any attempt at drawing coherent conclusions. However, recent efforts to pin down a critical period may bring clarity to this issue.

Let us turn to enrichment studies with bulb functional imaging as their endpoint. In one approach, adult mice were produced that carry a transgene that codes for a florescent signal correlated with OSN synaptic release. This allows bulb imaging for any OSN synapses innervating dorsal glomeruli. Using this method, one laboratory exposed mice carrying such a transgene to a week of intermittent passive odorant exposure or to one of two control environments [130]. The authors then imaged responses to the enrichment odorant, a similar odorant, and a dissimilar odorant. Enrichment narrowed the range of odorants that could trigger OSN transmitter release and reduced the quantity of transmitter released from responsive OSN terminals. In a subsequent study, the same laboratory imaged adult mice before and after seven days of intermittent passive odorant enrichment with an ester odorant or a home-cage control condition. The enrichment experience altered the temporal dynamics (but not the bulb’s spatial pattern) in response to a different odorant, an aldehyde, suggesting that the effects of enrichment are not odorant-specific [131].

Another approach is to use intrinsic signal optical imaging (IOS) to monitor odor-evoked bulbar signals, a technique that is also limited to dorsal glomeruli. In one such study, adult mice trained in an olfactory operant discrimination task with the odor pairs cineole/eugenol and ethyl butyrate/isoamyl acetate subsequently underwent IOS in an awake (head-fixed) protocol [132]. Compared to the naïve or passively enriched control groups, the trained group displayed more glomeruli activated by any of the enrichment odorants used as a stimulus. In addition, the glomeruli that were activated had greater signal intensity in the trained group. Why passive enrichment would trigger plastic responses in the formerly discussed study but not the latter is unknown [131,132].

Another study worthy of mention tried to create an actual “enriched” olfactory environment in the sense of Donald Hebb’s and Mark Rosenzweig’s (and colleagues) idea of studying the effects on the brain of richer and more stimulating environments than the norm [133,134]. In this study, adult mice were exposed for two hours per day for 21 days to a different complex natural odorant (e.g., cinnamon leaf oil, citral oil, etc.) each day [135,136,137]. Among other measurements, these authors examined the effects of enrichment on the IOS imaging of the bulb in anesthetized and awake mice. As in some other studies already discussed on passive single odor enrichment (see above), the authors found that, in anesthetized enriched mice, fewer glomeruli responded to the test odorants used compared to unenriched controls. However, responding glomeruli were larger, with stronger signals and more specificity compared to controls. Most of these results were qualitatively similar in the awake mice, if quantitatively less robust, such that some statistical differences were not significant. In addition, local field potential recordings showed that enrichment led to an increase in slow wave network activity (<12 Hz) and a decrease in fast oscillations (>12 Hz) compared to unenriched controls, though the significance of this result is unclear [135].

M/T neurons, the bulb’s primary outputs, have also been the focus of numerous enrichment studies. In one study, Pcdh21-Cre mice were virally transfected with a calcium-sensitive fluorophore to enable two-photon imaging of mitral cell activity [138]. Awake (head-fixed) mice were imaged before and after seven days of olfactory operant discrimination training (active enrichment) or passive odor enrichment. In both groups enrichment led to less discrete patterns of mitral cell activity (more overlap) for dissimilar odorants and more discrete (less overlap) patterns for similar odorants. The authors interpreted their results in terms of robust versus efficient coding mechanisms. Another study used a very similar calcium imaging protocol to study the effects of prenatal and early postnatal passive odor enrichment (in adulterated food) on mitral cell odor-evoked responses [139]. In contrast to the previous study, early food-based enrichment caused an increase in the amplitude, number, and reliability of mitral cell responses that were not specific to the enrichment odor. To explain this inconsistency with previously reviewed work, the authors suggested that prenatal enrichment may have been the critical factor in triggering the nonspecific effects of enrichment.

### 4.4. Olfactory Cortex

The piriform cortex (PCX), which is the main target of bulbar afferents, has been the primary focus of enrichment studies at the cortical level. While there have been relatively fewer studies, compared to the mucosa and bulb, there has been longstanding interest in the role of experience in PCX plasticity. As a quintessential auto-associative network, there was early recognition that PCX would likely be rapidly modified by experience [140]. Indeed, electrophysiological recordings from anterior PCX (aPCX) neurons in anesthetized rats revealed that as little as 50 s of odorant exposure caused habituation to occur that was not seen in the simultaneously recorded upstream bulbar neurons [141]. This duration of exposure to a binary odorant mixture was enough to allow aPCX neurons to differentiate the mixture from its components, unlike bulbar M/T cells [142]. Moreover, these brief odor experiences could foster olfactory perceptual stability and discrimination through the processes of pattern completion and pattern separation [143,144]. Subsequent single-unit recording studies in rat aPCX demonstrated that activity in this cortical component during the slow-wave state was influenced by recent odor experience, suggesting a role for these processes in odor memory consolidation [145].

The effects of odor enrichment (experience) on aPCX that have been reviewed so far were the results of painstaking electrophysiological studies. However, these findings also have been confirmed at a more holistic level through the visualization of Arc immediate early gene expression in pyramidal neurons of aPCX [146]. In this study, rats that had been trained in a go-no-go operant odor discrimination task subsequently had their aPCX responses visualized by Arc immunochemistry. The results document experience-dependent “sharpening, separation, and merging” in aPCX pyramidal cell ensembles.

As was the case in the mucosa and bulb, the role of passive versus “active” (associative) stimulus experience has been an issue of interest in olfactory cortical plasticity. Two studies in rats, using associative tasks with water rewards, have established task-dependent modifications in cortical odor relationships [144,146]. And a recent study using two-photon imaging of aPCX in transgenic reporter mice was able to show an effect of passive odor exposure on cortical odor representations [147]. The authors exposed mice to mixtures of aldehyde and ketones for 30 min three times per day for 14 days and then imaged cortical responses to individual components. Passive experience with mixtures led to a greater overlap of the cortical representations of individual components. The authors concluded that cortical representations of odor relationships are modifiable by the statistics of the odor environment.

To our knowledge, the effectiveness of passive and active odor experiences on cortical odor representations has not been rigorously compared, nor have there been any attempts to measure the duration of experience that is sufficient to modify cortical odor relationships. There is also a dearth of knowledge concerning the durability of these changes.

### 4.5. Neurogenesis

It is unclear whether odor enrichment increases neurogenesis in the olfactory mucosa. But, as noted previously, enrichment does lead to prolonged OSN survival [113]. As already mentioned in the deprivation section, the survival of adult-born neurons migrating into the bulb is decreased by olfactory deprivation [79]. The reverse is also true: olfactory enrichment increases the survival rate of bulbar adult-born neurons, which die otherwise at a surprisingly high rate (~60%) in “normal” olfactory environments [136,148]. Behavioral and physiological experiments suggest that the incorporation of adult-born neurons into bulbar circuits promotes certain types of olfactory learning and memory [149] as well as olfactory discrimination [150]. However, the role, if any, of adult-born neurons in olfactory behavior remains controversial [151]. In any event, a detailed review of this area is beyond our intended scope, though we will return to the concept in the theory section.

### 4.6. Behavior

**Figure 4:**
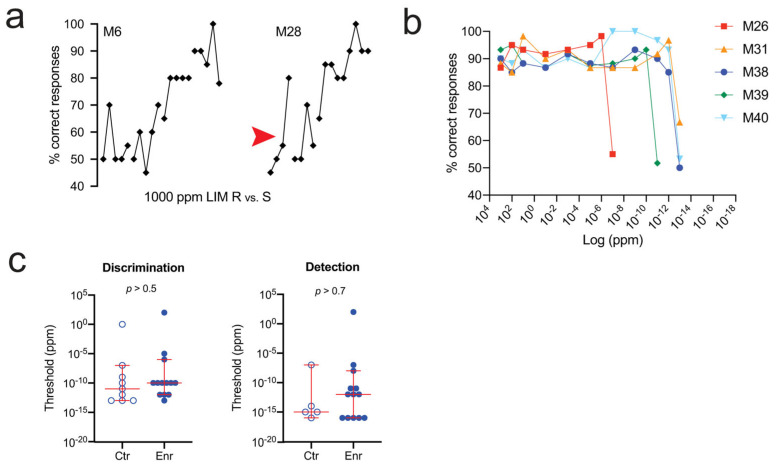
Psychophysical tests of limonene enantiomer from an operant task. Diamond symbols denote the percentage of correct responses for consecutive 20-trial blocks. Read the plot from left to right. Lines connect blocks from the same session. (**a**) Data from two mice naïve to odorants learning to discriminate 1000 ppm limonene (LIM) R vs. S. As was typical, mice made progress on the task within a couple of sessions. Mouse #28 (arrowhead) performed above chance after 40 trials. (**b**) Performance of five subjects discriminating decreasing dilutions of limonene progressing to the threshold (seen as a precipitous decline in percent correct). The discrimination task was S vs. R + S where R and S are two stereoisomers of limonene at 1000 ppm stock solution in mineral oil. Symbols are the percent correct for an individual mouse in the last two blocks of trials. (**c**) Threshold discrimination and detection for limonene. Mann–Whitney *p*-values are shown. Note: limonene enrichment did not affect median discrimination or detection thresholds (see text). Modified from [157].

Prior to Wysocki’s and colleagues’ discovery of olfactory induction (see above), there had already been considerable interest in the effects of odor enrichment on olfactory acuity [105]. In human psychophysical studies, positive effects of practice (e.g., lower thresholds) were common findings [152,153,154,155]. By contrast, early animal studies, which typically employed high odorant concentrations and long odor enrichment durations (months, in some cases), generally failed to detect any enrichment effect (reviewed by Cunzeman and Slotnick) [156]. Perhaps surprisingly, null results were even found after rats were exposed to enrichment odors from PND4 to PND70 and then threshold-tested with computer-controlled operant techniques [156]. Additionally, in a recent study of mice, no effect of enrichment was found using either active or passive enrichment (Figure 4) [157]. The author used a commercial olfactometer to measure discrimination and detection thresholds for limonene or carvone enantiomers before and after enrichment with the same compounds for up to three months. While the authors obtained some of the lowest thresholds ever measured for any species, they did not observe any effect of enrichment on either detection or discrimination.

Despite this body of null results, reports of significant effects of odor enrichment on olfactory behavior in animals abound in the literature. A few years after the induction phenomenon was demonstrated in animals using mouse EOG recordings (see above), the same laboratory demonstrated, also in mice, significant improvements in the behavioral threshold to androstenone and urine after enrichment with these odorants, suggesting that induction might be a general phenomenon [158]. In subsequent studies from the same laboratory, induction in mice was shown to last as long as 8 months after the enrichment had ceased [159].

In parallel with the aforementioned studies, there have been numerous reports of postnatal behavioral preferences induced toward enrichment odors delivered to mice, rats, or rabbits while in utero, typically by dams being fed odorant-adulterated chow during gestation [110,114,128,160,161].

Associative odor conditioning is a process by which enrichment has been shown to affect olfactory acuity. In one study of associative conditioning, using the cardiac orienting response to measure odor habituation and cross habituation in adult rats, olfactory aversive conditioning could improve the discrimination of similar odorants [162]. Perhaps the most surprising demonstration of associative conditioning’s effects on olfactory acuity provided strong evidence for transgenerational—presumably epigenetic—effects. In this study, an odor-potentiated startle response paradigm was used to condition the F0 generation of mice, conditioning that was subsequently shown to be transferred to the enrichment-odor-naïve F1 generation [120]. In another recent study, pairing rabbit maternal pheromone (unconditioned stimulus) with an arbitrary odorant (conditioned stimulus) in a single 5 min session induced heightened behavioral interest and EOG responses to the conditioned stimulus in newborn rabbits [112]. Judging by these studies, associative odor conditioning appears to have both rapid and long-lasting effects on olfactory acuity.

In comparison, non-associative or passive odor enrichment has been shown, repeatedly, to alter subsequent odor acuity. In one oft-cited study in adult rats, passive odor enrichment for one hour twice daily for 20 days improved subsequent odorant discrimination, as shown by habituation–dishabituation behavioral testing in rats [157,163]. In another study from the same laboratory, reminiscent of androstenone induction in anosmic humans, naïve rats that were unable to discriminate the enantiomers of limonene acquired this ability after being exposed to these odorants for 10 days. However, unlike classical induction, the effects of enrichment were not specific [164]: passive enrichment not only enabled the subject to discriminate the enrichment odors (limonene enantiomers) but also the other unrelated odorants that were tested. Similar results were subsequently confirmed in mice [131,137,165]. In another noteworthy recent study, one of the rare cases of true olfactory environmental enrichment, adult mice were exposed to 21 different natural or synthetic odorant blends in two one-hour sessions per day using a different odorant each day for 21 days [135]. Enriched mice were compared to non-enriched controls using habituation/dishabituation tests and a predator odor avoidance test. The results showed that odor enrichment improved the discrimination of an arbitrary set of odorants in the habituation/dishabituation test and the avoidance test.

By contrast, in another study of passive enrichment, mice were exposed to octanal from PND 10 through PND 31 either continually or intermittently (four times per day for 15 min each). Only the continually enriched group showed a significantly longer latency to find a vial of octanal in a modified “find the cookie” test. The authors interpreted this finding as consistent with the desensitization shown in the EOG component of the same study (see above) [115].

Given that both associative and non-associative enrichment can alter behavioral acuity, an obvious question that has rarely been tested is: which process is more effective? To address this question, one group of authors working with mice compared passive exposure using odorants delivered an hour per day for 10 days in the home cage to 20 trial sessions per day over 10 days of a forced-choice odorant discrimination task [166]. The behavioral testing was, as described above, a standard habituation–dishabituation task. The results were consistent with the conclusion that while both enrichment experiences improve spontaneous odor discrimination in a non-specific way, passive enrichment proved the more effective experience. Another study of mice compared learning rates in a computer-controlled go-left or go-right odor discrimination task [167]. Daily 30 min operant testing sessions were carried out in each of three groups: (1) additional operant training, (2) passive odor exposure, or (3) no supplemental experience (control group). The results were that additional operant training increased the rate of odor discrimination learning and that passive odor exposure was as effective as additional daily operant training in causing this improvement. A rather different result was found in a study of dogs that were either exposed to the enrichment odor passively or through Pavlovian conditioning [168]. Both exposure types lasted for five min and were repeated six times per day for seven days. Thresholds for enrichment and control odors were determined before and after enrichment using a go-no-go task controlled by a computer-assisted olfactometer. Dogs that were enriched by Pavlovian odor conditioning achieved a significant increase in sensitivity to the conditioned odor. There was no change in sensitivity in the group that was passively exposed to odors, nor was there any change in sensitivity to the control odor that was not used in enrichment. It is unclear why the rodent work has repeatedly shown the benefit of passive odor exposure (see above) but this study on dogs found no such effect. One possibility is differences in the concentration of the enrichment odor or simply a species difference.

We started this section on the behavioral effects of olfactory enrichment by citing some of the work that has shown a positive effect of practice in human subjects [152,153,154,155]. While it is beyond the scope of this review to discuss it in full, we will end the section by citing some of the clinical applications of olfactory enrichment. Various protocols of olfactory enrichment have been shown to improve olfactory abilities after nasal infection [169,170,171], after head trauma [171,172], among the aged [173] and in those suffering from Parkinson’s disease [171,174]. A meta-analysis of 13 such studies confirms significant effects of enrichment across diverse patient populations [171]. Results include significant positive effects on odor identification, discrimination, and threshold. Finally, in a very recent study [175], the effect of enrichment on learning was tested in a group of cognitively typical older adults. The subjects were exposed, while they slept, to 2 h per night of odor enrichment or a no-odor control condition. Odorants were delivered from a simple commercial odorant diffuser and odorant type was changed daily in a 7-day cycle over six months. Subjects, both enriched and unenriched controls, were administered a standard test of auditory verbal learning at the end of the study. Remarkably, a 226% improvement was seen in the enrichment group compared to the unenriched group. This study is notable because of the relative simplicity of the enrichment protocol and the size of the effect, demonstrating the potential and practicality of odor enrichment in the prevention and treatment of cognitive loss.

### 4.7. Theory

The theoretical grounds for studying the effect of odor enrichment on the olfactory system are manifold. Like the theoretical underpinnings of deprivation, Hebb’s ideas have been influential here, too. Though he did not study the brain per se, he did observe that rats raised as pets performed better at behavioral tasks than rats raised in cages [133]. Brain studies of “enriched” environments (i.e., rat playgrounds) eventually demonstrated their positive effects on synaptogenesis, cortical thickness, and other brain parameters that could explain the behavioral superiority of animals raised in enriched environments [176,177].

A problem inherent in these studies is defining what constitutes enrichment. As we have seen in olfaction, enrichment, in a vast number of studies, has been limited to exposure to one odorant at a high concentration. Given that there are >1000 olfactory receptor types in most of the experimental animals used in these studies, this kind of enrichment may have only a limited impact on neural activity, even accounting for the fact that many receptors are broadly tuned. This problem is compounded if odor exposure during enrichment remains constant or is intermittent but has a long (hours) exposure period, as both schedules would undoubtedly lead to OSN adaptation. However, we have reviewed studies whose authors have partially overcome this problem by creating the olfactory equivalent of the rat playground or have focused on one to two ORs and their cognate ligands!

Another justification for studying odor enrichment, or at least olfactory experience, is the phenomenon of perceptual learning: the enduring improvement in sensory acuity brought on by experience with the stimulus [178]. As we have seen, investigators in olfaction, like in other sensory realms, have been keen to show the effects of experience on the brain and behavior. We have reviewed several demonstrations of experience-dependent plasticity at the level of OSNs, the bulb, and the cortex. A notable finding, which resonates with studies of perceptual learning in other sensory systems, is the ability of stimulus exposure to “teach” with or without feedback to the subject. Indeed, some of the studies reviewed here have found passive (without feedback) odor enrichment to be as impactful or more impactful than active (with feedback) enrichment on olfactory structures, physiology, and behavior. Induction is a prominent example of plasticity without feedback that is, as already noted, at least partly rooted in OSNs.

Sparse neural coding is the representation of objects by the strong activation of relatively few neurons, which has, among other attributes, the quality of efficiency [179]. Enrichment with a single odorant, and thus a redundant signal in the environment, should, in theory, lead to a sparsening representation of that odor. Investigating this concept has been the motivation for the use of odor enrichment by several investigators [88,138].

In a related vein, the hypothesis that an ideal receptor array should not only match but predict the ever-changing odor environment has garnered some attention. As noted above, the use of odor enrichment and single-cell transcriptomics, in mice, has revealed unexpected OR specific transcriptomes that are modulated by odor environments. Thus, a group of genes coding for olfactory transductory elements and ORs appears to be regulated, up or down, by activity in order to match the statistics of the odor environment [29,30].

## 5. Conclusions

### 5.1. Overview

Nowhere has the search for and analysis of modes of plasticity been more energetic than in the olfactory system. Undoubtedly, this interest is explained, at least in part, by the fact that the system possesses two lifelong founts of new neurons, in the mucosa and sub-ventricular zone. Investigations of plasticity have been abetted by, as in studies of other sensory systems, the use of stimulus manipulation: deprivation and enrichment. While there have been some inspired experimental programs and seminal findings in this long quest, many questions remain, including about the validity of the methods themselves. What follows is a modest attempt to analyze the state of the field based on the literature we have reviewed.

### 5.2. Deprivation

As we have seen, UNO has been the mainstay of those seeking to apprehend the effects of stimulus impoverishment on olfaction. Though questions have been raised about some of the assumptions of UNO, its profound and myriad effects, particularly ipsilaterally, are well documented in the studies reviewed herein. There can be little doubt that the use of UNO has helped establish numerous instances of odor-dependent processes in both developing and adult mammals, although the latter dichotomy has blurred over time. And, as has been reviewed, these processes exist at all levels of the system from OSNs through the cortex. The extensive list of odor-dependent processes revealed using UNO includes many clearly deleterious effects establishing the indispensability of the stimulus for normal development and/or adult function, best understood from a Hebbian perspective.

However, we have also reviewed a more recent corpus that documents the salubrious effects of UNO. In these instances, reducing or, in the limit, eliminating the stimulus sets in motion processes, at all levels of the system, that appear to be homeostatic countermeasures. In certain cases, like for OSNs, compensatory responses to UNO have been cross-validated at the histochemical, electrophysiological, and transcriptomic levels. In the latter case, the effects of UNO on OSNs have recently been further confirmed by scRNAseq [29,30]. Collectively, these findings of compensatory responses to deprivation are easier to explain from an evolutionary perspective than deleterious effects where the logical necessity of activity dependence is often unclear either from an instructive or a permissive viewpoint.

### 5.3. Enrichment

Enrichment, which we have defined here as any provision of odorants by the investigator for the purpose of studying plasticity, has had a somewhat more dubious record than deprivation in studies of activity-dependent processes in olfaction. One area of success has been in the study of bulb-to-pyriform cortex code transformations. Several studies have documented that experience with the stimulus either in task-based or passive settings, over a range of time scales, can modify cortical odor relationships in a way that suggests adaptation to environmental odor statistics [144,146,147]. While relatively few odorants and mostly simple mixtures have been used in this research, so far, this work promises to reveal the nature of bulb-to-cortex transformations underlying odor-space to cortical-space coding.

By contrast, studies of activity-dependent processes in the mucosa and bulb, typically employing high concentrations of one or two odorants, have led to a spate of contradictory results. Though inconsistent findings are not unique to this field, the number and magnitude of the evidence at variance are surprising. To give but one example, odorant enrichment has been reported to increase OSN survival [113], decrease OSN survival [20], or not affect survival at all [127]. Rather than attempting an explanation for the disparate results, we thought it more productive to suggest some potential reasons for the lack of agreement among studies.

First, there are often problems with the independent variable: specifically, it is very difficult to compare the amount of enrichment across studies in this area since the actual concentration of odorants the subjects’ olfactory systems are exposed to is typically unmeasured and may be unmeasurable in some cases [130]. Thus, two different studies of the effect of, say, odor X may be using concentrations that are hundreds of folds different from each other.

Second, the studies we have reviewed vary widely in the timing of odor exposure, ranging from continuous exposure for months to a few seconds during electrophysiological recording. Rapid OSN adaptation in the case of continuous enrichment would be expected. However, given the high odor concentrations and long exposure durations often used in enrichment studies, the adaptation of OSNs may be predicted even with intermittent enrichment schedules. In such cases, it is far from clear how much enrichment-induced activity is occurring in OSNs. Indeed, we have been unable to find a single instance of the measurement of olfactory system activity during extended enrichment schedules. Third, a perennial problem in olfactory research concerns how to represent such a large stimulus space with a few odors. Most studies reviewed here used one or two enrichment odors selected from a quite limited overall pool. Such under-sampling, though completely understandable given the complexity of some of the experiments, almost certainly leads to a biased view of system functioning.

Problems with the dependent variable also accrue: First, cage-rearing (but differences in cage ventilation itself alters olfaction [35]) within a typical animal facility is such an impoverished sensory environment that any form of novel stimulus might simply have a ceiling effect in subjects. Second, as alluded to above, enrichment odors are often used at millions of times behavioral thresholds. This could cause very widespread adaptation (as already noted), inflammation, tissue damage, or systemic toxicity, whose effects are unpredictable and largely unstudied. Such effects might explain the null results from early studies using high concentrations of continuous odors to study the effects of enrichment on threshold [156]. Third, all but a few studies of the effects of olfactory enrichment on behavioral responses in animals have relied on the habituation/dishabituation technique. Habituation, a decrease in response to a stimulus upon repeated presentation, is a form of non-associative learning [180]. Typically, the test consists of presenting an odorized object for a minute or more and the time is noted for which the subject is judged to be sniffing the object. This procedure is repeated some number of times, noting on each trial the time the subject spends sniffing the object. A decline in investigation times with repeated trials is judged as a sign of odor recognition. If habituation occurs, odor discrimination can be tested in a final trial by presenting a novel odor. A significant increase in attending to the novel stimulus (dishabituation) is taken as evidence of the discrimination of the original and novel odor. While a more thorough discussion of the advantages and limitations of this method can be found elsewhere [181], briefly, the technique often results in an unreliable index of odor acuity because investigation times are typically judged subjectively, and it is difficult to maintain blind observations. Also, this measure is inherently weak since the subjects, by definition, are unmotivated. This test characteristic makes it hard to interpret negative results. These and other shortcomings doubtless explain why the test results often diverge greatly from more exacting olfactory tests. For example, it has been reported that naïve mice and rats cannot discriminate the enantiomers of limonene without several days of passive exposure to the compound, though operant methods show very rapid discrimination learning (beginning after a few trials) and a discrimination threshold in the parts per quintillion range [157,164,165].

Fourth, the EOG, a measurement frequently used to assess the effect of enrichment on the OSN responsivity, can be problematic. Though it is easy to perform and has no peer as a measurement of the bulk activity of OSNs, it is exceedingly difficult to obtain reliable quantitative data with this technique because of its inherent variability over time and space [82]. This may in part explain the conflicting results reviewed in which stimulation with the enrichment odor has variously caused an increase [182], a decrease [114,115], or no change [116] in the EOG amplitude.

### 5.4. Induction

**Figure 5:**
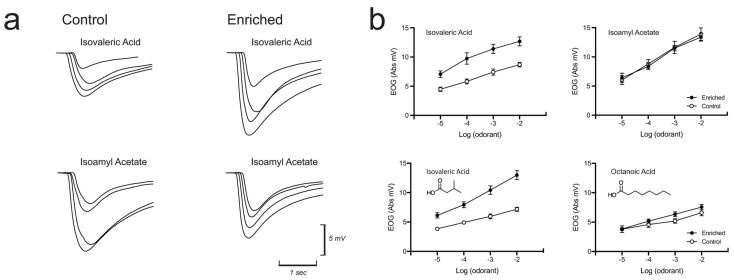
(**a**) EOG responses from C57BL/j mice that experienced an enrichment or control protocol. Stimuli were a log dilution series (10^−2^ to 10^−5^ M) of isovaleric acid (IVA; **upper** panel) or isoamyl acetate (IAA; **lower** panel). Left traces are the responses of a control subject. Right traces are the responses of subjects enriched with IVA daily for ≥2 weeks. Enriched subjects have larger amplitude EOGs for every concentration of IVA, while enrichment did not affect responses to IAA. (**b**) Means (±SEM) of the absolute values of EOG amplitudes from ten enriched and ten control subjects. The left plots show the data when IVA was the stimulus. Control and enrichment data are significantly different. The right plots show data from the same recording location when IAA (**upper**) or octanoic acid (OA; **lower**) was the stimulus. For these stimuli, responses from the control and enrichment groups were not significantly different. Modified from [86].

The topic of induction calls for special comment: key components of the classic experiments demonstrating inbred mouse strain induction have recently been replicated and extended (Figure 5) [86]. Considering key differences, these authors assert that olfactory induction in humans may only be superficially similar to the induction described in inbred mouse strains. Conversely, based on fundamental similarities, like the magnitude of effect and onset and recovery timing, these authors further proposed that mouse induction may have the same mechanism as compensatory plasticity, the homeostatic responses of OSNs to odorant deprivation. Though counterintuitive, it may be that both phenomena are controlled by internal cellular processes of OSNs that will only be understood when we have a more complete picture of olfactory peripheral adaptation and desensitization. If these speculations prove correct, induction, at least in non-humans, should not be viewed as a form of perceptual learning, the current view. Indeed, in the recent study mentioned, induction could not be produced in outbred mice after considerable effort with several odorants. Rather, it is suggested that induction is a secondary effect of the adaptation process in inbred strains that may lack adaptation-resistant OSN subtypes. Thus, deprivation and enrichment could engage identical pathways in OSNs whose evolved function is to normalize sensory activity in the face of environmental instability.

### 5.5. Neurogenesis

Though we have not reviewed many studies of adult-born olfactory neurons, we will comment on the intersection of this phenomenon and odor enrichment. The conventional explanation holds that life-long neurogenesis, unique to olfaction, affords the system an ability to adjust to newly encountered olfactory environments throughout the lifetime [131,183,184]. However, there are reasons to question this hypothesis. First, olfactory receptor expression has been shown to be surprisingly stable over an organism’s lifetime and across related taxonomic groups over evolutionary time. For example, Khan and colleagues [185], using NanoString technology, found only ~4% of olfactory receptor transcripts were differentially expressed during a mouse’s lifespan. Meanwhile, Furudono and colleagues [186] found that the physiologically measured odorant response groupings of mice and the psychophysically measured odorant quality groupings of humans were similar. This suggests conservation in olfactory receptors despite the taxonomic and ecological chasm between mice and humans. Second, as we have already seen, deprivation or enrichment have ambiguous effects on OSN proliferation, causing increases in some cases and decreases in other cases [27,116].

What is the function of adult-born neurons in olfaction? “Gain-of-function” studies link them to improvements in detection and discrimination [187,188], memory [150], perceptual learning [183], and innate behaviors [150,189]. However, “loss-of-function” studies have, for the most part, been unable to demonstrate any major effects on olfaction by inhibiting neurogenesis [151,190]. For example, the human rostral migratory stream is highly curtailed beyond the embryonic period, and yet our species’ olfactory abilities are quite respectable [191,192].

Finally, we lack any obvious explanation as to why other sensory systems manage to adapt to new surroundings without a constant supply of adult-born neurons [193].

Taken together, these facts suggest to us an alternative view of adult-born neurons in olfaction. As we have explained in detail elsewhere [13], we propose that adult neurogenesis in the olfactory system may not be a new adaptation for organisms to adjust to the vagaries of the odor milieu. Rather, this characteristic of OSNs and bulbar inhibitory neuron progenitors may be a vestige of an ancestral neotenic state that has been maintained for the purposes of ongoing repair in this, the only sensory system whose sensory cells make direct contact with the unforgiving environment [13].

### 5.6. Aspirations

The plethora of technological advances such as scRNAseq, two-photon microscopy, and high-resolution MRI, to name a few, assures that some dependent variables in studies of stimulus-dependent olfactory plasticity will be measured with increasing precision. We propose that the same technological sophistication should now be applied to the independent variable: odor enrichment. Consider that most of the studies we have reviewed used nothing more sophisticated than a tea ball (egg) placed on the home cage top to deliver enrichment odors in animal studies. Also, we could not find a single study that had anything approximating an accurate measure of odor concentration during odor enrichment at the subjects’ nares, much less any measure of activity in the olfactory system engendered by the enrichment condition. We suggest that the era of using single, high-concentration, monomolecular odorants in enrichment studies should end given all the discrepant results this approach has wrought. The field needs detailed measurements and simulations of actual odor environmental statistics, though this aspiration will clearly challenge current analytical tools.

While odor deprivation in the form of naris occlusion can be reasonably standardized across laboratories (ignoring the issue of the time of occlusion and opening), a further challenge is the multitude of odor enrichment procedures used, regarding the type(s) of odor used, the type of odor source, duration, and frequency of odor application (intermittent or continuous) to name a few. Standardization across laboratories would be helpful, while probably also unachievable.

Perhaps the most critical dependent variable of all, behavioral acuity measures, also deserves additional comment. We have opined on the shortcomings of the habituation/dishabituation test, still the predominant method for measuring behavioral acuity in olfactory research. We suggest that more exacting methods like odor-conditioned taste avoidance, aversive conditioning, and operant conditioning should find expanded use. These methods have their own shortcomings but should be used to confirm findings from non-associative methods like habituation/dishabituation.

Despite the 150 years that have transpired since the first study of how the stimulus affects olfactory plasticity was performed, we still lack clear answers to some of the most fundamental questions. Breakthroughs, in our view, are going to require keen attention to the stimulus, not just the plasticity.
